# Metabolic Rewiring in Adult-Type Diffuse Gliomas

**DOI:** 10.3390/ijms24087348

**Published:** 2023-04-16

**Authors:** Jong-Whi Park

**Affiliations:** 1 Department of Life Sciences, College of BioNano Technology, Gachon University, Seongnam 13120, Republic of Korea; jpark@gachon.ac.kr; Tel.: +82-(0)32-899-6115; 2 Department of Health Sciences and Technology, GAIHST, Gachon University, Incheon 21999, Republic of Korea; 3 Neuroscience Research Institute, Gachon University, Incheon 21565, Republic of Korea

**Keywords:** glioma metabolism, isocitrate dehydrogenases (IDH), therapeutic strategies

## Abstract

Multiple metabolic pathways are utilized to maintain cellular homeostasis. Given the evidence that altered cell metabolism significantly contributes to glioma biology, the current research efforts aim to improve our understanding of metabolic rewiring between glioma’s complex genotype and tissue context. In addition, extensive molecular profiling has revealed activated oncogenes and inactivated tumor suppressors that directly or indirectly impact the cellular metabolism that is associated with the pathogenesis of gliomas. The mutation status of isocitrate dehydrogenases (IDHs) is one of the most important prognostic factors in adult-type diffuse gliomas. This review presents an overview of the metabolic alterations in IDH-mutant gliomas and IDH-wildtype glioblastoma (GBM). A particular focus is placed on targeting metabolic vulnerabilities to identify new therapeutic strategies for glioma.

## 1. Introduction

Cellular metabolism generates energy (ATP), biosynthetic precursors, cofactors, reducing equivalents (NADPH and NADH), and macromolecules, such as nucleic acids, proteins, and lipids, which are crucial for biological functions. As a result, the metabolic pathways in normal and malignant cells play a pivotal role in cell growth and survival. Experimental studies have shown that altered cellular metabolism supports sustained proliferation and enhances tumor development and progression [1]. Increased glycolytic activity is required for the rapid division of cancer cells, leading to glucose accumulation in glycolytic tumor tissues [2]. To find evidence that cellular metabolism contributes to pathogenesis, non-invasive metabolic screening has been developed, including 18F- fluorodeoxyglucose positron emission tomography (FDG-PET) [2]. Deregulating cellular energy metabolism is now recognized as a fundamental hallmark of cancer, as proposed by Hanahan and Weinberg [3].

Normal and transformed cells that are proliferating rely on glycolysis instead of oxidative phosphorylation (OXPHOS) and produce lactate from glucose, even in the presence of adequate oxygen, known as the Warburg effect [4,5]. Unlike the intense dependence on glycolytic metabolism in growing and dividing cells, most differentiated cells use OXPHOS for ATP production rather than glycolysis [6]. It has been established that rapidly dividing cells undergo metabolic adaptation without mitochondrial function defects [7]. For example, the inhibition of lactate efflux adversely affects the glioma invasion, indicating that accelerated lactate efflux is required for invasive abilities in tumor cells [8]. In addition to the invasive phenotype, glycolytic metabolism can regulate and delay programmed cell death [9].

Gliomas are thought to originate from glial or precursor cells. Several lines of evidence reveal significant differences in the metabolic profiles between isocitrate dehydrogenase (IDH)-mutant gliomas and IDH-wildtype glioblastomas [10,11,12]. For example, increased reactive oxygen species (ROS) are detected in IDH1-mutant cells [10]. Additionally, IDH1-mutant gliomas rely on oxidative phosphorylation, while IDH1-wildtype gliomas primarily rely on glycolysis for ATP production [11,12]. Similarly, a patient-derived oligodendroglioma xenograft model showed an increased mitochondrial activity compared to IDH1-wildtype xenografts [13]. This review presents the metabolic differences between IDH-mutant and IDH-wildtype gliomas. Furthermore, it reviews the novel therapeutic strategies that target metabolic alterations in preclinical and clinical studies.

### 1.1. Glioma

Gliomas account for 80% of malignant brain tumors [14] and have been classified and graded based on histological features [15]. However, this has resulted in inter-observer variability and minimal clinical efficacy. Despite the standard treatments, including surgical resection and chemoradiotherapy, the clinical outcomes have been unfavorable over the previous decades due to the development of drug resistance and tumor recurrence [15]. Next-generation sequencing provides information on genetically distinct alterations in adult and pediatric gliomas [16,17,18,19]. For example, mutations in IDH and histone variants frequently occur in adult and pediatric gliomas, respectively [15]. Oligodendroglioma is characterized by the co-deletion of chromosome arms 1p and 19q (1p/19q codel) along with IDH mutations (Table 1), while astrocytoma is 1p/19q non-codel with loss of alpha-thalassemia/mental retardation syndrome X-linked (ATRX). BRAF is the most commonly altered molecular driver in pediatric low-grade gliomas [20], with BRAF V600E mutations being more frequent in pleomorphic xanthoastrocytoma (PXA) and ganglioglioma (GG) [20,21].

### 1.2. Glycolysis

The brain consumes approximately 25% of the glucose in the human body [22]. Specifically, astrocytes rely on glycolytic pathways, while neurons exhibit higher rates of the oxidative metabolism [23]. The brain uses alternative energy substrates such as lactate and ketone bodies in harsh environmental conditions. Cells take up glucose via specific glucose transporters (GLUTs), and hexokinase (HK) converts glucose to glucose-6-phosphate, which is the first step in glucose metabolism. HK1 is highly expressed in normal brain and low-grade gliomas, whereas HK2 is overexpressed in developing embryos and glioblastoma (GBM) tissue [24,25]. A study by Wolf and colleagues suggested that the demethylation of HK2 intron1 triggers its expression in human GBM [25]. The pentose phosphate pathway (PPP) is closely linked with glycolysis and is essential for NADPH regeneration synthesis [26].

The last committed step in glycolysis is the conversion of phosphoenolpyruvate to pyruvate, which is catalyzed by pyruvate kinase. Variable expressions and heterozygous mutations of pyruvate kinase type M2 (PKM2) have been reported in human breast tumors [27]. Similarly, PKM2 is upregulated in GBM specimens, and the siRNA-mediated downregulation of PKM2 leads to decreased levels of ATP and glutathione [28]. Mechanistically, PKM2 interacts with β-catenin upon the stimulation of the epidermal growth factor (EGF), and PKM2-dependent β-catenin transactivation is required for GBM development [29]. A similar but independent study reported that PKM2 physically interacts with Histone H3, and PKM2-dependent phosphorylation is essential for cell proliferation and tumor growth [30].

### 1.3. Mitochondrial Metabolism

The mitochondrial pyruvate carrier (MPC), which consists of two proteins, MPC1 and MPC2, is essential for efficient pyruvate uptake in the inner mitochondrial membrane. Impaired mitochondrial transport has been observed in many cancers, including gliomas. In particular, mitochondrial pyruvate carrier1 (MPC1) is under-expressed in gliomas, which is correlated with reduced survival in patients [31]. It has also been demonstrated that MPC restoration forms a mitochondrial complex and reduces tumor growth in vivo [31]. Interestingly, high MPC1 expression is strongly associated with a better prognosis in IDH-mutant and 1p/19q codel gliomas, but not GBM [32].

Other mitochondrial enzymes involved in oxidative metabolism include pyruvate dehydrogenase (PDH), which irreversibly converts pyruvate into acetyl-CoA. PDH is phosphorylated and inactivated by pyruvate dehydrogenase kinase (PDK), leading to decreased pyruvate oxidation in mitochondria and accelerated lactate production in the cytosol. The activity of PDH can be increased by PDH phosphatase (PDP) expression, which is repressed in patient-derived GBM samples [33]. In line with this, PDP1 restoration reduces GBM tumor growth [33].

Acetyl-CoA combines with oxaloacetate to form citrate. Citrate can be exported from the mitochondria and cleaved by ATP-citrate lyase (ACLY) to generate acetyl-CoA for fatty acid synthesis and histone acetylation. Within the mitochondria, several reactions enable citrate to be decarboxylated to oxaloacetate, producing CO_2_ and converting nicotinamide (NAD) and flavin adenine dinucleotides (FAD) to NADH and FADH2. These reducing equivalents are oxidized in the mitochondrial electron transport chain (ETC) to generate an electrochemical gradient, which is necessary for ATP synthase. Interestingly, mutations in the mitochondrial complex III and IV, ETC components, have been found in GBM [34]. Cytochrome c oxidase (complex IV) and ATP synthase (complex V) in the ETC can be repressed by D-2-hydroxyglutate [35,36].

### 1.4. Glutamine Metabolism

**Figure 1:**
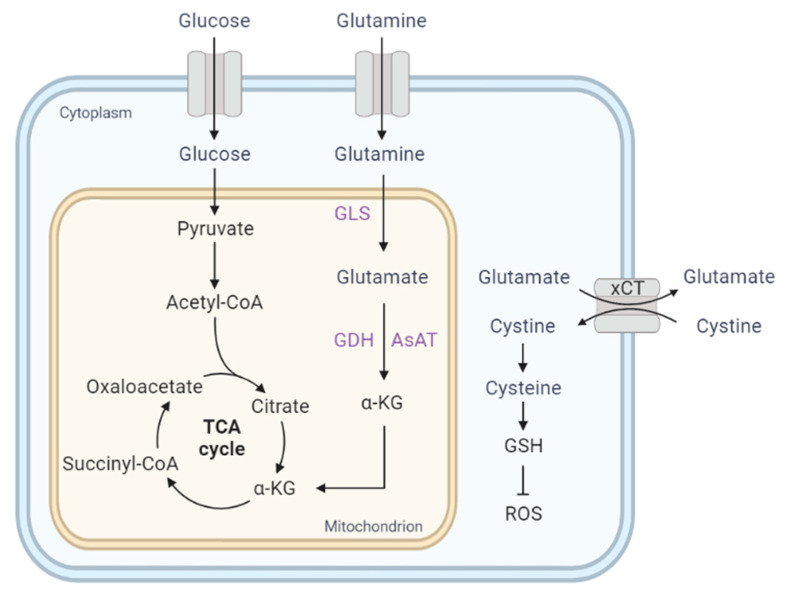
Glutamine metabolism supplies TCA cycle intermediates (anaplerosis). The cystine/glutamate antiporter xCT (SLC7A11) promotes extracellular cystine uptake for glutathione biosynthesis. α-KG, α-ketoglutarate; AsAT, aspartate aminotransferase; GDH, glutamate dehydrogenase; GLS, glutaminase; GSH, glutathione; ROS, reactive oxygen species.

Glutamine metabolism serves as a nitrogen and carbon source for the biosynthesis of nucleotides and amino acids and it can replenish the carbon backbone as an anaplerotic substrate for the tricarboxylic acid (TCA) cycle function. Additionally, glutamine metabolism supports NADPH production for fatty acid synthesis. During glutaminolysis, glutamine is first converted to glutamate by glutaminase (GLS). Glutamine-derived glutamate can be metabolized by glutamate dehydrogenase (GDH) to produce α-ketoglutarate (α-KG) for maintaining cellular homeostasis (Figure 1). Glutamate can also be converted to glutamine by glutamine synthetase (GS), which is an astrocytic enzyme.

Transformed cells exhibit a high rate of glutamine metabolism during rapid proliferation [37], and increased glutamine uptake within tumors has been observed in human gliomas but not in normal brains [38]. ASCT2 (Slc1a5), the key glutamine importer, is strongly expressed in a rat astrocytoma-derived glioma model [39]. It has been demonstrated that MYC-dependent metabolic alteration or glutaminolysis renders cells addicted to glutamine for protein and nucleotide biosynthesis [40], and consequently, the transaminase inhibitor aminooxyacetate (AOA) induces selective toxicity in MYC-transformed cells [40]. In glutamine-starved GBM cells, GS sustains de novo purine biosynthesis [41].

Glutamine metabolism also promotes drug resistance to mTOR kinase inhibitors in an α-KG-dependent manner [42]. This compensatory upregulation of GLS and glutamate by mTOR inhibitors confers survival advantages [42]. It has been shown that the pharmacologic inhibition of mTOR kinase promotes cystine uptake and glutamate secretion via the cysteine/glutamate antiporter (xCT) encoded by the SLC7A11 gene [43]. Particularly, cysteine uptake is important for glioma cells to maintain cellular redox balance by producing glutathione (GSH) [43] (Figure 1). Gu et al. found that mTORC2 specifically binds to xCT, reducing xCT activity by phosphorylation on serine 26 [43]. In addition, xCT is responsible for glioma-mediated neuronal toxicity via the glutamate release [44,45]. Importantly, the treatment of sulfasalazine (SAS), an FDA-approved xCT inhibitor, reduces peritumoral glutamate in glioma patients [44]. Like the glutamine importer, glioma cells upregulate the expression of glutamatergic receptors, such as NMDA and AMPA [46,47,48]. Interestingly, IDH-wildtype gliomas utilize glutamine and glucose for metabolic pathways, whereas IDH1-mutant gliomas depend on glutamate and lactate [49]. This is discussed further in the section on metabolic reprogramming in IDH-mutant glioma.

### 1.5. Lipid Metabolism

Fatty acid and cholesterol biosynthesis are essential for the basic structure of cellular membranes in proliferating tumor cells. The evidence shows that fatty acids can cross the blood–brain barrier (BBB) via fatty acid transport proteins [50]. In contrast, dietary cholesterol cannot enter the central nervous system due to the BBB. The evidence suggests that GBM cells depend on cholesterol metabolism and exhibit selective vulnerability to liver X receptor (LXR) ligands [51]. Moreover, GBM with constitutively active epidermal growth factor receptor (EGFR) signaling is particularly susceptible to the depletion of sterol regulatory element-binding protein 1 (SREBP-1), which is a transcription factor for fatty acid and cholesterol synthesis [52]. A recent study reported that triglycerides (TG) are prominently formed in GBM tissues and are required for GBM survival via autophagy or glucose deprivation-mediated TG hydrolysis [53]. Kant and colleagues demonstrated that fatty acid β-oxidation (FAO) plays a central role during gliomagenesis [54]. Comprehensive metabolic profiling of patient-derived gliomas revealed that the biological function of FAO depends on the diverse tumor microenvironment [54]. Surprisingly, the antidepressant fluoxetine inhibits sphingomyelin phosphodiesterase 1 (SMPD1), the key enzyme for sphingolipid biosynthesis, and tumor progression in patient-derived GBM orthotopic xenograft models [55]. Consistent with this finding, clinical observations show that fluoxetine significantly prolongs the survival of GBM patients [55].

## 2. Metabolic Reprogramming in IDH-Mutant Glioma

Under physiological and pathological conditions, IDH enzymes play an important role in cellular metabolism as a critical component of the TCA (also known as the citric acid or Krebs cycle). IDH1 and IDH2 use nicotinamide adenine dinucleotide phosphate (NADP+) as a cofactor to generate NADPH, as illustrated in Figure 1 [56]. NADPH is vital for buffering ROS and lipid metabolism [57,58]. IDH1 is a cytosolic NADP+-dependent metabolic enzyme that mediates the oxidative decarboxylation of isocitrate to produce α-ketoglutarate (α-KG) or 2-oxoglutarate (2-OG). Additionally, IDH is essential for antioxidant defense through glutathione recycling [59].

Isocitrate dehydrogenase (IDH) mutations are prevalent in astrocytoma and oligodendroglioma [60,61]. IDH2, which is an analog of IDH1, is predominantly localized in mitochondria but mutated in gliomas at much lower frequencies [62]. IDH3 is also a mitochondrial enzyme that uses NAD+ instead of NADP+. Hotspot mutations of IDH1/2 have been identified in various human malignancies, including diffuse gliomas, myelodysplastic syndrome (MDS), chondrosarcoma, intrahepatic cholangiocarcinoma (ICC), and acute myeloid leukemia (AML) [17,61,63,64,65,66]. IDH1 mutations work in tandem with other oncogenic events to promote astrocyte proliferation and glioma development [67,68]. After a telomere-induced crisis, IDH mutations induce telomerase reverse transcriptase (TERT) reactivation, which is linked to astrocyte immortalization and transformation [69].

**Figure 2:**
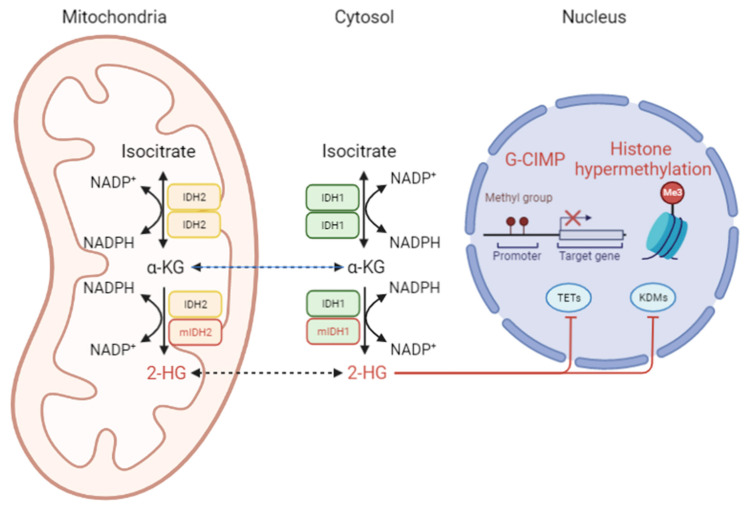
The pathophysiological role of the IDH enzyme. As homodimers, IDH1 or IDH2 produce α-KG in the cytosol or mitochondria, respectively. The prevalence of the IDH mutation is high in adult-type low-grade gliomas. mIDH gains a neomorphic enzymatic activity, leading to 2-HG accumulation. Consequently, 2-HG competitively binds to ten-eleven translocation (TET) enzymes and histone lysine demethylase (KDMs). 2-HG, D-2-hydroxyglutarate; α-KG, α-ketoglutarate; G-CIMP, glioma-CpG island methylator phenotype; H3, KDM, histone lysine demethylase; mIDH1/2, mutant IDH1/2; TET, ten-eleven translocation protein.

Heterozygous mutations at codon R132 or R172, respectively, lead to the production of D-2-hydroxyglutate (2-HG) with neomorphic enzyme activity in IDH1 and IDH2 (Figure 2) [66,70]. Moreover, IDH mutations significantly impact components of the TCA cycle intermediates and amino acids [71]. In IDH1- or IDH2-mutant cells, the levels of tyrosine, serine, threonine, methionine, tryptophan, phenylalanine, asparagine, and glycine are increased, while glutamate, aspartate, and N-acetylated amino acids are depleted [71]. Due to the limited capacity to metabolize 2-HG, intracellular 2-HG can accumulate up to 30 mM and impede α-KG as an antagonist [70]. The production of 2-HG alters redox metabolism and triggers oxidative stress in cell culture conditions, leading to the dependency of IDH-mutant cells on exogenous lipid sources [72]. The mitochondrial production of proline is enhanced to maintain redox homeostasis in IDH1-mutant glioma cells [73].

2-HG affects the α-KG-dependent dioxygenases family of enzymes, including the TET family of DNA hydroxylases and JumonjiC (JmjC) domain-containing histone lysine demethylases (KDMs) (Figure 2) [74,75]. Recently, 2-HG-induced overexpression of stearyl-CoA desaturase (SCD) was found to cause significant alterations in the phospholipids and morphology of the endoplasmic reticulum (ER) and Golgi [76]. IDH1-mutant glioma lines are more susceptible to oleic acid-induced apoptosis than wildtype counterparts [76]. Fack et al. showed that orthotopic patient-derived xenografts of IDH-mutants exhibit remarkable differences in phospholipid composition, reduced glucose turnover, and lower energy potential [11]. These authors further revealed that cystathionine-β-synthase (CBS) is highly expressed in IDH-mutant gliomas, and its expression correlates with prolonged survival in oligodendroglioma patients [11]. 2-HG also inhibits 2-OG-dependent branched-chain amino acid (BCAA) transaminases (BCATs) and glutamate synthesis, increasing glutaminase dependence [77]. McBrayer and colleagues demonstrated that the GLS inhibitor CB-839 impairs cell proliferation by depleting glutathione, particularly in IDH1-mutant HOG cells under oxidative stress but not in their IDH-wildtype counterparts [77]. Interestingly, Zaprinast, a phosphodiesterase type 5 inhibitor, reduces the levels of 2-HG and inhibits the glutaminase (GLS) enzyme in IDH1-mutant cells [78].

An earlier study showed that the prolyl hydroxylase domain (PHD)-containing enzymes are inhibited by 2-HG treatment [74]. However, unlike the initial observation, several studies have shown that 2-HG promotes prolyl hydroxylase activity, leading to low levels of the hypoxia-inducible factor subunit HIF-1alpha in IDH-mutant gliomas [79,80]. IDH-mutant tumors grow slowly under hypoxic conditions in vivo, which is possibly due to altered metabolic consequences [12]. In particular, increased OXPHOS and decreased glutamine metabolism are observed in IDH1-mutant cells. As a result, the selective inhibition of glutaminase slows down IDH-mutant cells [81]. Conversely, the overexpression of glutamate dehydrogenase 2 (GLUD2) rescues the growth-inhibitory effect of IDH1 mutation in murine glioma progenitor cells [82].

## 3. Metabolic Reprogramming in IDH-Wildtype Glioblastoma

Due to its highly aggressive nature, GBM was the first tumor type to be sequenced by The Cancer Genome Atlas [16]. More than 90% of GBMs exhibit genetic aberrations in the RTK/RAS/PI3K pathway [16]. Constitutive Akt activation is sufficient to promote glucose consumption, exhibiting high rates of glycolysis [83]. GLUT3 is highly expressed as a glucose transporter, particularly in classical and proneural GBM subtypes, via PAK4-YAP/TAZ signaling [84]. Additionally, shRNA-mediated knockdown targeting integrin β3 strongly reduces the expression of GLUT3, glucose uptake, and lactate production [84]. Phosphatase and tensin homolog (PTEN) mutations are associated with high levels of HK2, enabling GBM cells to proliferate in a distinctly unique microenvironment [24].

As one of the major downstream targets of the PI3K/AKT signaling pathway, the mammalian target of rapamycin (mTOR) signaling is dysregulated in many cancers, including glioblastoma. The experimental evidence demonstrates that Nf1 deficiency promotes astrocyte proliferation in an mTOR-dependent manner [85]. It is becoming increasingly apparent that α-KG or 2-HG inhibits ATP synthase and mTOR signaling [35,36,86]. IDH-wildtype GBM cells selectively produce 2-HG under hypoxic conditions [87]. Intlekofer et al. reported that lactate dehydrogenase A (LDHA) profoundly affects hypoxia-induced 2-HG [87].

EGFR gene amplification is found in about half of GBM patients [16], frequently harboring the EGFR gene rearrangement-induced constitutively activated mutant, EGFRvIII. It is becoming evident that the EGFRvIII mutation promotes glycolytic gene expression via MYC-dependent tumor cell metabolism [88]. Delta MAX, a truncated MAX protein, enhances the glycolytic gene expression and tumorigenic potential in EGFRvIII GBM cells [88]. MYC activation induces the expression of genes involved in glycolysis and glutaminolysis [89]. Using metabolic imaging in an orthotopic xenograft mouse model, Mair and colleagues found that lactate labeling positively correlates with the c-MYC-mediated expression of HK2, monocarboxylate transporters, and lactate dehydrogenase A (LDHA) [90]. Patient-derived GBM lines activated by MYC show glucose dependency, and these cell lines are selectively responsive to glycolytic inhibition with nicotinamide phosphoribosyl-transferase (NAMPT) inhibitors [91].

p53 balances glycolysis and oxidative phosphorylation, reducing ROS for cell survival under normal conditions. Under severe metabolic stress, p53 also has a pro-oxidant activity that can remove impaired cells [92]. Mai et al. found that cytoplasmic p53 is required for erlotinib-induced apoptosis. The combined targeting of EGFR-driven glucose utilization and pharmacological p53 stabilization suppresses tumor growth in orthotopic GBM xenograft models [93]. It is important to note that Costunolide-induced ROS production has selective toxicity, particularly in glioma cells A172 and the U87MG bearing wildtype p53, but not in p53-mutant T98G [94]. TERT regulates PPP and glycogen accumulation [94]. The TERT-promoter mutations C228T and C250T are commonly found in GBM and oligodendroglioma [95]. TERT-promoter mutant GBM tumors show high fatty acid synthase (FASN) levels and lipid accumulation [96]. In addition, TERT downregulation by TERT siRNA results in the decreased expression of peroxisome proliferator-activated receptor gamma co-activator 1-alpha (PGC-1α) [96].

## 4. Crosstalk of Metabolic and Epigenetic Signaling in Glioma

The interplay between epigenetic modifications and metabolic alterations affects tumor cell heterogeneity and plasticity in gliomas. For example, the forced expression of Enhancer of zeste homolog 2 (EZH2), the catalytic subunit of the polycomb repressive complex 2 (PRC2), enhances glycolytic metabolism instead of mitochondrial respiration [97]. In addition, HIF1α is required for EZH2-mediated metabolic adaptation in gliomas [97]. MiR-215 is post-transcriptionally induced by HIF1α bound to Drosha/DGCR8 complexes under hypoxia [98]. Interestingly, the inhibition of MiR-215 attenuates the sphere-forming ability and the tumorigenic capability of glioma stem cells [98]. The lysine methyltransferase G9a and G9a-like protein (GLP) methylate HIF-1α at K674 and inhibit its transcriptional activity under hypoxia [99]. Importantly, G9a is downregulated by chronic hypoxic conditions in GBM [99].

As described above, SREBP-1 plays a central role in lipogenesis. Glucose enhances the stability of SREBP cleavage-activating protein (SCAP) by N-glycosylation, leading to SREBP-1 activation [100]. In this sense, EGFR signaling can trigger SREBP-1 activation via increasing the glucose uptake [100]. On the contrary, defects in the N-glycosylation of SCAP reduce the orthotopic tumor growth in GBM-bearing mice [100]. As a negative feedback regulator, miR-29 expression is induced by the SCAP and SREBP-1 complex, and subsequently, miR-29 inhibits SCAP and SREBP-1 by targeting their 3′-untranslated region (3′-UTR) [101].

Kelch-like ECH-associated protein 1 (KEAP1) plays a crucial role in the ubiquitin-mediated degradation of NF-E2-related factor 2 (NRF2) [102]. In many human cancers, somatic mutations of KEAP1 or NRF2 disrupt the interaction of these two proteins, consequently leading to the accumulation of NRF2 and upregulation of its target genes, even under stressed conditions [102]. It has been reported that KIAA0132, the human homolog of INrf2, inhibits the ubiquitin–proteasome pathway for NRF2 degradation [103].

## 5. Tumor Microenvironment

Nutrient availability in the tissue context and cell-autonomous mechanisms, such as genomic alterations and oncogenic signaling, can modulate metabolic needs in brain tumors. Recent investigations have demonstrated that low tumor oxygenation, also known as hypoxia, correlates with glioma cell spreading and worse patient survival [104]. Lactate efflux into the extracellular space creates an acidic tumor microenvironment (TME), resulting in drug resistance and blocking the cytotoxic function of T cells [105]. Low oxygen tension also alters the levels of NAD and NADP, enhancing the catabolism of proteins to fulfill the bioenergetic demand [106]. A recent study showed that monocyte-derived macrophages are more abundant in IDH-wildtype gliomas, while microglia are enriched in IDH-mutant gliomas [107].

2-HG inhibits complement activation in a dose-dependent manner and diminishes complement-mediated phagocytosis [108]. T cells can uptake tumor cell-derived 2-HG via the sodium-dependent dicarboxylate transporter 3 (SLC13A3), leading to impaired T-cell antitumor immunity [109]. It is therefore becoming apparent that a decreased number of infiltrating T cells is observed in IDH-mutant gliomas [107,108,109]. Additionally, 2-HG inhibits T-cell proliferation, migration, and cytokine secretion [108]. The computational characterization approach found that MHC-I subunit human leukocyte antigen (HLA) genes are significantly methylated in IDH-mutant glioma cell lines compared with IDH-wildtype GBM lines, suggesting that the MHC-I-mediated antigen presentation is impaired in IDH-mutant gliomas [110].

## 6. Therapeutic Approaches for Targeting Metabolic Vulnerabilities

**Figure 3:**
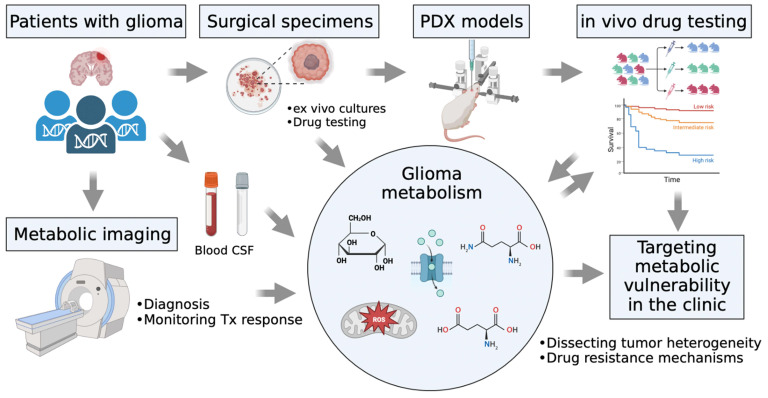
Metabolic profiles of glioma characterized by plasma and the cerebrospinal fluid (CSF) of the patients, patient-derived cell lines, intracranial mouse models, and preclinical drug testing.

Altered cell metabolism in cancer cells offers metabolic vulnerabilities that could be exploited therapeutically. Thus, a comprehensive understanding of glioma metabolism involved in tumor heterogeneity and drug resistance mechanisms can target metabolic vulnerability and translate into the clinic to benefit patients (Figure 3). As a glycolytic inhibitor, 2-deoxy-D-glucose (2-DG) has been shown to potentiate radiation-induced ER stress in GSCs [111]. 2-DG also protects normal brain tissue from radiation damage in the clinic [112]. Dietary restrictions have been shown to sensitize gliomas to radiation therapy [113]. An in silico super-enhancer screen identified ELOVL Fatty Acid Elongase 2 (ELOVL2) as critical for GBM stem cell proliferation [114]. The combined targeting of EGFR signaling and polyunsaturated fatty acid synthesis displays a synergistic effect on glioma stem cells (GSCs) [114]. Although the mTOR inhibitor rapamycin has not been effective in the clinic, ATP-competitive mTOR kinase inhibitors CC214-1 and CC214-2 provide promising therapeutic efficacy in orthotopic xenografts [115]. Gini and colleagues found that a preferential effect of CC214-1 is more pronounced in glioma cells with EGFRvIII expression and PTEN loss [115]. Importantly, the combined inhibition of mTOR kinase and glutaminase (GLS) profoundly reduces tumor growth in a GBM xenograft model [42].

The accumulated evidence suggests that maintaining 2-HG levels or IDH1 mutations may not be essential for glioma growth, as the loss of 2-HG or mutant IDH1 expression alone is insufficient to prevent tumor propagation [116,117]. Interestingly, IDH mutations or the epigenetic effect of 2-HG can decrease the level of NAD+, and inhibiting nicotinamide phosphoribosyltransferase (NAMPT) can induce cytotoxicity in endogenous IDH1/2-mutant cancer cells [116]. Additionally, the increased enzymatic activity of SIRT1, an NAD+-consuming enzyme, in combination with NAMPT inhibition, can enhance the antiproliferative effect in patient-derived IDH-mutant cells [118].

GSCs show reduced mitochondrial respiration, and they are resistant to conventional alkylating chemotherapy drugs, such as temozolomide (TMZ) and 1,3-bis(2-chloroethyl)-N-nitrosourea (BCNU) [119]. The glycolytic inhibition mediated by 3-bromo-2-oxopropionate-1-propyl ester (3-BrOP), along with carmustine (BCNU), shows a dramatic effect in killing GSCs [120]. Ritonavir (RTV), a non-specific GLUT antagonist, shows low BBB permeability [121]. Dual treatment with RTV plus BCNU increases the overall survival in the GL261 murine tumor model [121]. Interestingly, high glucose transporter isoform 1 (GLUT1) expression is detected in quiescent endothelial cells [122]. Surprisingly, the loss of GLUT1 in endothelial cells impairs brain angiogenesis in vivo without altering BBB physical functions [122].

Glioma cells utilize both glycolysis and mitochondrial oxidation in vivo [123]. Pharmacological inhibition of mitochondrial components, mutant IDHs, and lipids is also being tested in ongoing clinical studies (Table 2). Dichloroacetate (DCA) induces ROS production, showing antitumor and antiangiogenic effects in C6 glioma cells in vivo [124]. As a pyruvate dehydrogenase inhibitor, DCA penetrates the BBB and normalizes the mitochondrial functions in three of five GBM patients [125]. Mechanistically, DCA contributes to p53 activation and mitochondrial ROS generation [125]. Oliva et al. found that mitochondrial DNA is susceptible to damage by sustained TMZ treatment [126]. In addition, the pharmacological and genetic intervention of cytochrome c oxidase (COX) restores TMZ sensitivity in TMZ-resistant glioma cells [126]. The small molecule IACS-010759, an inhibitor of mitochondria complex I of the ETC, reduces tumor growth in mouse models of brain cancers and acute myeloid leukemia (AML) [127].

Ivermectin, an antiparasitic drug, markedly suppresses GBM tumor growth via mitochondrial respiration inhibition [128]. Given that tumor hypoxia is a key contributor to radioresistance, targeting tumor hypoxia by antiparasitic agents (ivermectin, proguanil, mefloquine, quinacrine, and atovaquone) is a promising approach for enhancing radiosensitivity [129]. Imipridone, which is also called ONC201, suppresses glucose metabolism and OXPHOS-dependent ATP production in stem-like GBM cells [130]. High levels of oxaloacetate have been shown to reduce glioma growth in animal models [131], and anhydrous Enol-Oxaloacetate (AEO) is being utilized to evaluate clinical response. A recent study reported that targeting GLS enzymatic activity by CB-839 eradicates stem-like GBM cells [132]. Based on the preclinical finding, CB-839, combined with radiation and temozolomide, is being utilized in phase 1 clinical trial of IDH-mutant astrocytoma (Table 2; ClinicalTrials.gov NCT03528642).

## 7. Conclusions

Oncogene-directed reprogramming provides a unique metabolic adaptation to anabolic growth or elevated rate requirements. On the other hand, unique dependencies expose metabolic vulnerabilities that can be exploited as a metabolically targeted therapeutic approach. However, several challenges need to be considered, such as the permeability of the BBB, glioma heterogeneity, and the dynamic tumor microenvironment, particularly in terms of the nutrient and oxygen availability. An improved understanding of metabolism in normal physiology and brain tumors is needed to revolutionize the treatment of these intractable diseases. Given that alternative therapeutic options can be combined with epigenetic drugs or immunotherapy, further studies are required to tackle heterogeneous glioma metabolism.

## Figures and Tables

**Table 1:** Molecular-based classification of adult-type diffuse gliomas.

Classification	IDH Status	Molecular Profile	WHO Grade
Oligodendroglioma	IDH mutation	Chromosome 1p/19q codeletion	2/3
Astrocytoma	IDH mutation	1p/19q non-codeletion	2/3
		CDKN2A/B homozygous deletion	4
Glioblastoma	IDH wildtype	TERT-promoter mutation	4
		EGFR amplification	4
		Gain of chromosome 7/Loss of chromosome 10	4

**Table 2:** Ongoing clinical trials of metabolic agents in adult-type diffuse gliomas.

Drug	ClinicalTrials. Gov Identifier	Phase	Cancer Type	Enrollment	Primary Outcome Measure (s)	Sponsor (s)
Metformin	NCT04945148	2	IDH wildtype GBM	640	Progression-Free Survival (PFS) by the RANO	Hopital Foch
Metformin	NCT04691960	2	GBM	36	Tolerability of metformin	Weill Medical College of Cornell University
Metformin	NCT05183204	2	GBM	33	Progression-Free Survival (PFS) by the RANO	Weill Medical College of Cornell University
Metformin	NCT02780024	2	GBM	50	Overall Survival (OS)	McGill University Health Centre
Metformin	NCT01430351	1	GBM	144	Safety and tolerability	M.D. Anderson Cancer Center
Dichloroacetate (DCA)	NCT05120284	2	GBM	40	Efficacy (ObsRO)	University of Florida
ONC201	NCT03295396	2	H3 K27M-mutant glioma	95	Overall response rate	Chimerix
Telaglenastat (CB-839)	NCT03528642	1	IDH mutant astrocytomas	40	MTD and/or RP2D	National Cancer Institute (NCI)
BAY1436032	NCT02746081	1	IDH1 mutant solid tumors	81	MTD and/or RP2D	Bayer
AG-120 AG-881	NCT03343197	1	IDH1 mutant gliomas	49	2-HG concentration	Institut de Recherches Internationales Servier
AG-881 (Vorasidenib)	NCT04164901	3	IDH1/2 mutant gliomas	340	Progression-Free Survival (PFS)	Institut de Recherches Internationales Servier
AG-120 (Ivosidenib)	NCT02073994	1	IDH1 mutant solid tumors	170	Safety and tolerability, MTD, and/or RP2D	Institut de Recherches Internationales Servier
IDH305	NCT02381886	1	IDH1 mutant malignancies	166	The incident rate of dose limiting toxicities (DLTs)	Novartis Pharmaceuticals
TVB-2640	NCT03032484	2	Astrocytoma	24	Tumor response per RANO	The University of Texas Health Science Center in San Antonio

Abbreviation: MTD, Maximum Tolerated Dose; RP2D, Recommended Phase II Dose.

## Data Availability

Not applicable.
